# Pathophysiology and Neuroprotective Strategies in Hypoxic-Ischemic Brain Injury and Stroke

**DOI:** 10.3390/brainsci7080110

**Published:** 2017-08-22

**Authors:** Bruno P. Meloni

**Affiliations:** Perron Institute for Neurological and Translational Sciences; Centre for Neuromuscular and Neurological Disorders, The University of Western Australia; Department of Neurosurgery, ,Sir Charles Gairdner Hospital, QEII Medical Centre, Nedlands 6009, Australia; bruno.meloni@perron.uwa.edu.au; Tel.: +61-8-64570304

Hypoxic-ischemic brain injury and stroke are closely related and devastating conditions that can affect individuals of all ages. Acute brain injury following cerebral ischemia as a result of stroke or hypoxia-ischemia, includes perinatal hypoxic-ischemic encephalopathy (HIE) and global cerebral ischemia (e.g., cardiac arrest/resuscitation, vasospasm, cerebral edema) together are one of the major causes of death and disability worldwide. For example, stroke alone affects an estimated 16 million people worldwide annually [1]. Similarly, HIE affects 1–3 and up to 10–26 per 1000 infants born in developed and developing countries respectively [2,3,4]. An additional dimension is that most survivors of stroke and hypoxic-ischemic brain injury are left with serious life-long physical and/or neurological disabilities.

Despite considerable research over many years, there are still no proven clinically effective pharmacological neuroprotective therapies capable of reducing the severity of brain injury following stroke or cerebral hypoxia-ischemia. As a consequence, the development of an effective pharmacological neuroprotective agent for individuals suffering a cerebral ischemic event remains an urgent unmet need. To make matters worse, there seems to be a widespread sentiment among some researchers, clinicians and pharmaceutical companies that continuing research focused on the development of neuroprotective agents will ultimately be met with failure at the clinical level. This sentiment reflects the fact that past trials on neuroprotective agents have failed to produce a clinically effective agent. While it goes without saying that investing time and effort in the development of an effective neuroprotective agent is high risk, the potential benefits in terms of improving patient outcomes are enormous; hence most impartial observers would agree that the benefits of the continuing search for neuroprotective agents far outweigh the risks.

In terms of strategies to increase the likelihood of success in developing a clinically effective neuroprotective agent there are a number of lines of investigation that could be explored. First and foremost are further experimental and clinical studies to improve our understanding of the pathophysiological processes involved in ischemic brain injury in order to identify new therapeutic targets. Importantly, given that many of the pathophysiological events associated with stroke and hypoxic-ischemic brain injury are still not fully elucidated, there is great potential for such studies to identify a new set of potential therapeutic targets leading to the development of agents with better prospects for effective translation into the clinical arena.

An alternative, but complementary approach, is to identify new compounds or improve the efficacy of existing experimental agents with neuroprotective properties. For example, our laboratory has recently highlighted the potential of a class of peptides known as cationic arginine-rich peptides (CARPs) as potent neuroprotective agents, with demonstrated efficacy in vitro and in animal models of stroke [5,6,7,8,9,10,11] and HIE [unpublished data]. Furthermore, another consideration in order to provide the best opportunity for success in terms of obtaining efficacy at the clinical level, is assessment of combination treatments or identification of compounds with multiple mechanisms of action targeting two or more neurodamaging and/or neuroprotective pathways. To this end, CARPs are known to reduce neuronal calcium influx and excitotoxic neuronal death [6,7,11], down-regulate calcium channel and TNF receptor proteins [12,13,14,15], target and assist in maintaining mitochondrial integrity [16,17,18,19], reduce the activity of the proteasome [20,21] and inhibit proprotein convertases that activate matrix metalloproteinases [22,23]. In addition, this class of peptide has the capacity to modulate immune responses [24,25,26,27] and activate pro-survival signalling pathways [28,29]. 

Ultimately, a better understanding of the pathophysiology of ischemic and hypoxic brain injury and the identification of novel therapeutic targets and neuroprotective compounds will be an essential pre-requisite for the development of new and effective neuroprotective therapies. Hence the aim of this Special Issue is to encourage the publication of new experimental and clinical findings to advance our understanding of pathogenic processes and to identify novel neuroprotective strategies for hypoxic-ischemic brain injury and stroke. 
